# C/EBPα regulates the fate of bone marrow mesenchymal stem cells and steroid-induced avascular necrosis of the femoral head by targeting the PPARγ signalling pathway

**DOI:** 10.1186/s13287-022-03027-3

**Published:** 2022-07-26

**Authors:** Ping Duan, Hanyu Wang, Xinzeyu Yi, Hao Zhang, Hui Chen, Zhenyu Pan

**Affiliations:** Department of Orthopedics Trauma and Microsurgery, Zhongnan Hospital of Wuhan University, Wuhan, 430071 China

**Keywords:** SANFH, C/EBPα, PPARγ, Acetylation, Adipogenic differentiation

## Abstract

**Background:**

The imbalance of osteogenic/adipogenic differentiation of bone marrow mesenchymal stem cells (BMSCs) is closely related to steroid-induced avascular necrosis of the femoral head (SANFH). We aimed to investigate the epigenetic mechanism of intramedullary fat accumulation and continuous osteonecrosis after glucocorticoid (GC) withdrawal in SANFH.

**Methods:**

An SANFH model was established in SD rats, which received an intermittent high GC dose for the first 4 weeks followed by an additional 4 weeks without GC. We explored the synergistic effects and mechanisms of C/EBPα and PPARγ on the differentiation of BMSCs by lentivirus-mediated gene knockdown and overexpression assays. A chromatin immunoprecipitation assay was performed to identify epigenetic modification sites on PPARγ in vivo and in vitro.

**Results:**

In the SANFH model, intramedullary fat was significantly increased, and the transcription factors C/EBPα and PPARγ were upregulated simultaneously in the femoral head. In vitro, C/EBPα promoted adipogenic differentiation of BMSCs by targeting the PPARγ signalling pathway, while overexpression of C/EBPα significantly impaired osteogenic differentiation. Further studies demonstrated that histone H3K27 acetylation of PPARγ played an important role in the epigenetic mechanism underlying SANFH. C/EBPα upregulates the histone H3K27 acetylation level in the PPARγ promoter region by inhibiting HDAC1. Additionally, inhibiting the histone acetylation level of PPARγ effectively prevented adipogenic differentiation, thus slowing the progression of SANFH.

**Conclusions:**

Our results demonstrate the molecular mechanism by which C/EBPα regulates PPARγ expression by acetylating histones and revealed the epigenetic phenomenon in SANFH for the first time.

**Graphical abstract:**

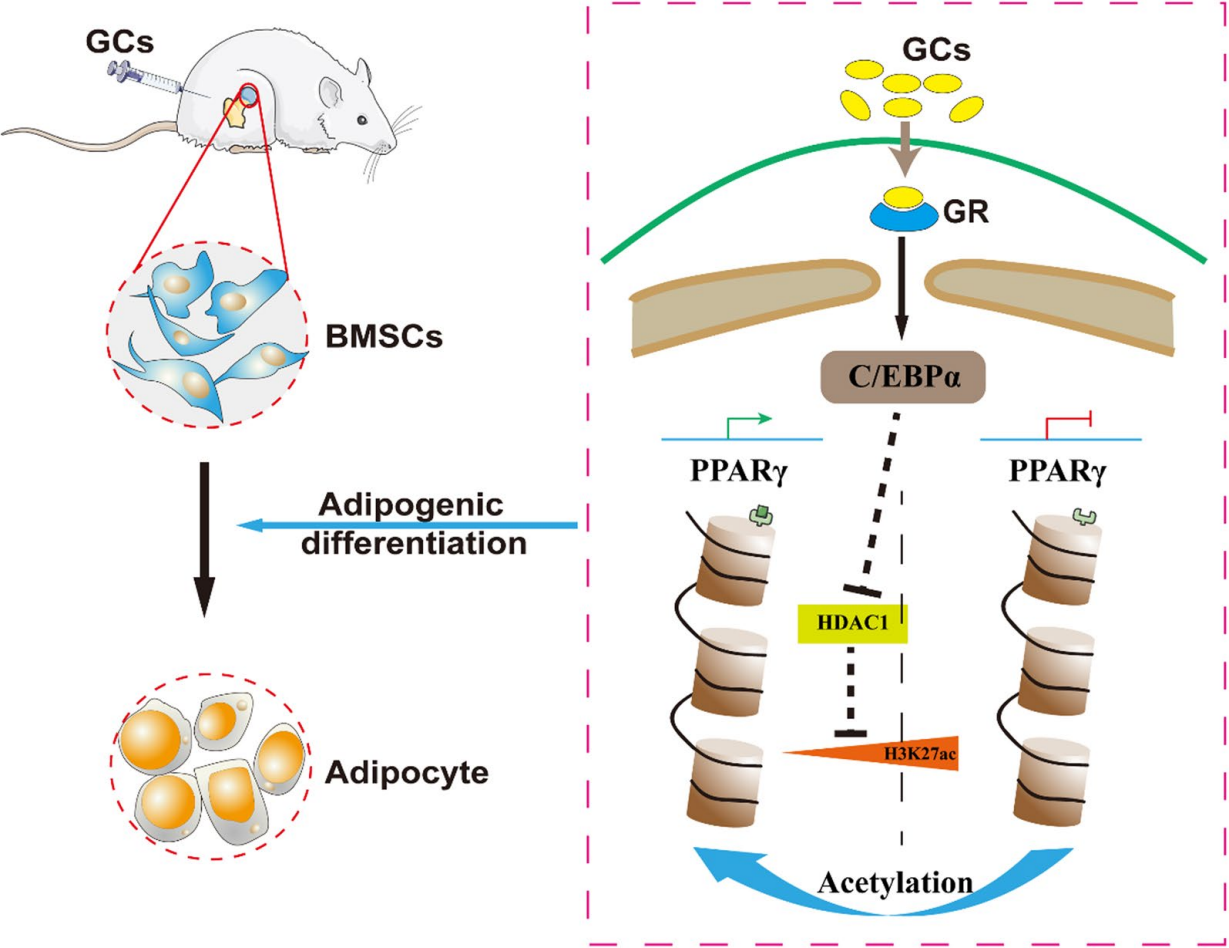

**Supplementary Information:**

The online version contains supplementary material available at 10.1186/s13287-022-03027-3.

## Introduction

Steroid-induced avascular necrosis of the femoral head (SANFH) is a progressive metabolic disease caused by the use of glucocorticoids (GCs) [1, 2]. To date, however, the exact pathological process of SANFH is still unclear, which may be considered the outcome of multiple factors, including apoptosis of bone and osteoblasts [3–5], prolonged survival of osteoclasts [6], abnormal coagulation activity [7], apoptosis of endothelial cells and disorder of vascular regeneration [8, 9], as well as the accumulation of fat in bone marrow and the rise of intraosseous pressure [10–12], eventually leading to the death of cells in the femoral head due to ischaemia and hypoxia, subchondral collapse and necrosis of the femoral head. There are many theories about the pathogenesis of SANFH [13], but very little information was found in the literature on the relationship between the molecular mechanism of SANFH and adipogenic differentiation.

Bone marrow mesenchymal stem cells (BMSCs) are capable of multidirectional differentiation. The imbalance of osteogenic/adipogenic differentiation in BMSCs may be an important mechanism of SANFH for the impairment of bone repair ability and intramedullary fat accumulation [14–16]. There is evidence that the peroxisome proliferator-activated receptor γ (PPARγ) signalling pathway plays a crucial role in regulating adipogenic differentiation of BMSCs and has been involved in the pathogenesis of SANFH [17–19]. In addition, GCs also play an important regulatory role in the proliferation and differentiation of BMSCs [20]. Glucocorticoid receptor (GR) is widely distributed throughout the body [21], and GCs can specifically bind to GR and recruit the coactivator transcription factor CCAAT/enhancer-binding protein α (C/EBPα) [22, 23]. Studies have shown that the C/EBPs transcription factor family (C/EBPα, β, δ) mainly regulates adipogenesis by assisting in regulating the expression of adipogenic genes and affecting the uptake of glucose by adipocytes. C/EBPβ and C/EBPδ initiate lipogenic signals at the early stage of lipid differentiation and then decrease rapidly, whereas C/EBPα persists steadily throughout [24, 25]. Many studies have confirmed the importance of C/EBPα and PPARγ in adipogenic differentiation [26, 27]; however, the specific regulatory mode of C/EBPα on PPARγ has not been deeply explored. In the SANFH model, the decreased bone mass and increased marrow fat tissue demonstrated that GCs can disrupt the normal differentiation of BMSCs, which accelerates adipogenesis but not osteogenesis [28]. However, it is still unclear why necrosis of the femoral head continues to progress in most patients after GC withdrawal [29].

Epigenetic modifications, mainly including DNA methylation, chromatin remodelling (such as histone methylation and acetylation), and genomic imprinting, are effective tools for studying the interaction between environmental signals and the genome. It has previously been observed that some genetic and epigenetic modifications of the genome are involved in the onset of SANFH, especially changes in the PPARγ regulatory domain, which are associated with an increased risk of SANFH [30, 31]. Data from several studies suggest that the inactivation of histone deacetylase 1 (HDAC1) is required for glucocorticoid-dependent preadipocyte differentiation [23, 32]. The acetylation level of histone H3K4/H3K9/H3K27 in PPARγ is positively correlated with PPARγ expression in the process of adipogenesis, and the expression of PPARγ was increased after histone H3K9ac in the PPARγ enhancer region was significantly increased [33, 34]. During adipogenic differentiation of 3T3-L1 cells, histone H3K9ac and H3K27ac of the PPARγ gene are significantly elevated, and increased acetylation of both promotes increased expression of PPARγ and maintains adipogenesis [35]. Therefore, we deduce that the histone acetylation of PPARγ may play a key role in SNAFH and adipogenic differentiation of BMSCs.

In the present study, we explain in detail that SANFH may be a disease related to the abnormal differentiation of BMSCs. In the SANFH model of Sprague Dawley (SD) rats, histone H3K27 acetylation in the PPARγ promoter region is an important mechanism of femoral head necrosis. Consistently, the specific regulatory mechanism of C/EBPα on the PPARγ signalling pathway in adipogenic differentiation of BMSCs was verified in vitro. Blocking the histone acetylation of PPARγ effectively inhibited adipogenesis and prevented the progression of SANFH without affecting the upstream effect of GCs.

## Methods

### Animal model of SANFH

Fifty-seven adult female SD rats (8–10 weeks old, weighing 220–250 g) were purchased from SIPEIFU Biotechnology Co., Ltd. (Beijing, China). All animals were normally fed by professional animal managers in the animal experiment centre of Wuhan University according to standard conditions. The environment is equipped with an air filtration system, and the animals can move freely in the cage.

After feeding for one week, the animals were accurately weighed and classified by cage (3 rats/cage), with 21 rats each in the control group and model group and 15 in the treatment group. SD rats in the model and treatment groups received intraperitoneal injections of lipopolysaccharide (LPS, E. coli 0111: B4, Sigma–Aldrich, China) twice, 20 μg/kg each time, with an interval of 24 h. Then, methylprednisolone sodium succinate (40 mg/kg, Pfizer, China) was injected into the buttocks of rats at an interval of 24 h each time, three times a week for 4 weeks. Subsequently, the rats in the model group were fed a normal diet for another 4 weeks after stopping the administration of GCs, whereas the treatment group received intragastric administration of curcumin (100 mg/kg, Sigma–Aldrich, China) three times a week for 4 weeks. Rats in the control group were treated with physiological saline, and the dose, times and duration were consistent with the above. In addition, the height of all feeding troughs was raised throughout the rearing period, thereby forcing the rats to stand up for food.

### Tissue samples

All rats were killed by pentobarbital (100 mg/kg) overdose 8 weeks after receiving the first treatment. Before the surgical operation, all surgical instruments were sterilized and were soaked with 0.1% diethyl pyrocarbonate to remove RNA enzymes, and the femoral head was obtained by surgical stripping and stored at −80 ℃.

### Microcomputed tomography (micro-CT)

To verify the necrosis of the femoral head in different groups, the samples were scanned at 8 μm resolution, 90 kV and 180 μA by a micro-CT system (SkyScan1276, Bruker, Belgium). After scanning, we constructed a 3D structure of the femoral head for analysing and comparing trabecular bone parameters. It mainly includes bone volume/tissue volume (BV/TV), bone surface area/tissue volume (BS/TV), the number of trabeculae (TB. N), trabecular thickness (TB. Th), trabecular separation (TB. Sp) and bone mineral density (BMD), which are quantified to determine the relative bone mass in the femoral head.

### Haematoxylin and eosin (H&E) staining and immunohistochemistry (IHC)

The femoral head was fixed in 4% paraformaldehyde solution (pH 7.4) for 3 days, decalcified and embedded in paraffin, cut into 5-μm-thick sections along the coronal plane with a slicer, and finally subjected to H&E staining. The remaining femoral head sections were dewaxed, antigen recovered, incubated with the primary antibody (rabbit polyclonal anti-type 1 collagen (COL1) and anti-PPARγ, Proteintech Group, Inc.), and then incubated with the appropriate horseradish peroxide-coupled secondary antibody. Finally, the sections were stained with DAB and counterstained with haematoxylin. The Leica Aperio VERSA 8 was used to obtain micro-scanning photographs. At least three different multiple visual fields were randomly selected for each slice in each group. Each slice was analysed by Image-Pro Plus 6.0 (Media Cybernetics, Inc., Rockville, MD, USA). The IHC results were defined as integrated optical density (IOD).

### Isolation and culture of BMSCs

BMSCs were extracted from SD rats aged 2–4 weeks. Bilateral femurs were isolated by aseptic operation, and the bone marrow cavity was rinsed with complete medium (Cyagen Biosciences, Inc., cat. no. RASMX-90011) to obtain a whole bone marrow suspension. Impurities were filtered with a 100-μm cell filter screen, inoculated in a 25 cm^2^ culture flask at a density of 4 × 10^4^ live cells/cm^2^, and maintained in a humidified environment with 5% CO2 at 37 °C. BMSCs with high purity were isolated by the whole bone marrow adherent method [36], and the cells after the third generation were used in subsequent experiments.

### Identification of BMSCs

BMSCs were identified by flow cytometry. A rat mesenchymal stem cell surface labelling detection kit (cat. no. RAXMX-09011) was purchased from Cyagen Biosciences, Inc. According to the manufacturer’s instructions, third-generation BMSCs were used for detection. Positive cell surface markers included CD44, CD90, CD29 and CD73; negative surface markers included CD34, CD11b/c and CD45; homotypic control antibodies against CD44, CD34, CD73, CD90 and CD45 were mouse IgG1; and homotypic control antibodies against CD11b/c and CD29 were mouse IgG2a and hamster IgG, respectively.

### Induction of osteogenic and adipogenic differentiation

BMSCs were inoculated into 6-well plates and cultured in specific SD rat osteogenic induction medium (Cyagen Biosciences, Inc., cat. no. RASMX-90021). The solution was changed every 3 days. After 21 days, alizarin red staining was performed to detect calcium deposits. The final results were quantitatively analysed by using ImageJ. To induce adipogenic differentiation, a specific SD rat adipogenic induction medium (Cyagen Biosciences, Inc., cat. no. RASMX-90031) was used. The induction medium and maintenance medium were used alternately for 16 days followed by continuous use of the maintenance medium for 4 days until the lipid droplets became large enough. Oil red O staining was used to detect adipocytes.

### Lentivirus infection

After repeated preliminary experiments, it was found that the effect of plasmid transfection of BMSCs was poor. Therefore, lentivirus infection was selected to overexpress C/EBPα (OE-C/EBPα) and PPARγ (OE-PPARγ) in BMSCs, and rat C/EBPα (NM_012524) and PPARγ (NM_013124) were cloned. Short hairpin RNA (shRNA) was expressed with lentivirus to knock down the expression of C/EBPα and PPARγ. The shRNA sequences targeting C/EBPα were as follows: shC/EBPα-1#5'-GTGCGCAAGAGCCGAGATAAA-3', shC/EBPα-2# 5'-GCCTGAGAGCTCCTTGGTCAA-3' and shC/EBPα-3# 5'-CCCTCACTTGCAGTTCCAGAT-3'. The shRNA sequences targeting PPARγ were as follows: shPPARγ-1#5'-GAGGGCGATCTTGACAGGAAA-3', shPPARγ-2#5'-AACCATCCGATTGAAGCTTAT-3' and shPPARγ-3#5'-CAGCATTTCTGCTCCACACTA-3'. All the above lentiviruses were prepared by Shanghai GeneChem Co., Ltd.

### Reverse transcription quantitative polymerase chain reaction (RT–qPCR)

Total RNA was extracted using a PureLink™ RNA Mini Kit (Invitrogen, 12183018A) according to the manufacturer's instructions. The effect of endogenous genomic DNA was removed by specific DNase (Thermo Scientific, K2981), and then cDNA was synthesized by a RevertAid RT reverse transcription kit (Thermo Scientific, K1691). Finally, mRNA was quantified by quantitative real-time PCR using a SYBR Colour qPCR mixture (Vazyme, Jiangsu, China) and a BIORAD CFX96Touch real-time PCR system. The PCR primer sequences of collagen type I, alpha 1 chain (COL1a1), alkaline phosphatase, biomineralization associated (ALP), C/EBPα, PPARγ and housekeeping gene β-actin (ACTB) are shown in Additional file 1: Data 1. Relative mRNA was calculated using the comparative 2^−ΔΔCt^ method.

### Protein extraction and western blot analysis

The femoral head protein needs to be fully ground in liquid nitrogen before extraction, but not for cell samples. Then, the cells were placed in RIPA buffer (Beyotime, P0013B) supplemented with phenylmethylsulfonyl fluoride (PMSF, Beyotime, ST506) and phosphorylated protease inhibitor (Servicebio, G2007) and lysed on ice for 30 min. The protein sample concentration (Absin, abs9232) was detected by the BCA method. Finally, an appropriate amount of SDS–PAGE protein loading buffer (Biosharp, BL511B) was added and boiled for 5–10 min. The same amount of protein was loaded and separated on an SDS–PAGE gel and then transferred onto a polyvinylidene fluoride (PVDF) membrane. After blocking in TBST containing 5% bovine serum albumin for 1.5 h at room temperature, the membrane was incubated with primary antibody at 4 ℃ overnight. The primary antibodies included rabbit anti-β-actin (Abcam, ab227387, 1:6000), anti-C/EBPα (CST, 2295S, 1:1000), anti-HDAC1 (Bio-Swamp, PAB36508, 1:1000), and anti-COL1a1 (CST, 91144S, 1:1000) and mouse monoclonal anti-PPARγ (Abcam, ab41928, 1:1000), anti-ALP (Santa Cruz, SC-365765, 1:1000) and anti-Runx2 (Santa Cruz, SC-390715, 1:1000). After washing, the PVDF membrane was incubated with the corresponding horseradish peroxide-conjugated secondary antibody (Goat anti rabbit or Goat anti mouse) at room temperature for 1 h. β-actin or histone 3 was employed as a loading control. The visualization of protein bands was carried out by ECL reagent (Biosharp, BL523A).

### Chromatin immunoprecipitation (ChIP) analysis

A total of 50–100 mg tissues or 2 × 10^7^ cells were fixed and cross-linked with 1% paraformaldehyde at room temperature for 15 min, and 1.25 M glycine was used to terminate the cross-linking. After precooling PBS was used to clean tissues or cells, the tissues were ground into powder under liquid nitrogen, where the cells formed clumps. The cells were lysed with cell lysis buffer (50 mM Tris–HCl, pH 8.0, 150 mM NaCl, 5 mM EDTA, 1% NP40) and centrifuged at 9000 × g for 5 min to obtain nuclear microspheres. Nuclear lysis buffer (50 mM Tris–HCl, pH 8.0, 50 mM NaCl, 5 mM EDTA, 1% Triton X-100) was used to lyse the nucleus. DNA was broken by ultrasound with a cycle of 1 s on and 5 s off at 20% power for 10 min, causing breakage into fragments between 200 and 500 bp. Then, samples were centrifuged at 9000×*g* for 5 min, and the nuclear lysate was incubated with protein A/G Agarose (Santa Cruz) at 4 ℃ for 1 h. Then, the supernatant was incubated with 5 μg antibody at 4 ℃ overnight. The next day, protein A/G agarose was added to the mixture and incubated at 4 ℃ for 2 h. After cleaning, the magnetic beads were resuspended in elution buffer. RNase A and proteinase K were added for digestion. Then, the samples were incubated at 65 ℃ for 6–10 h for decross-linking. The DNA obtained by ChIP was purified by a FastPure® Gel DNA Extraction Mini Kit (Vazyme, Nanjing). Subsequently, Taq Pro Universal SYBR qPCR Master Mix (Vazyme, Nanjing) was used to detect the target sequence.

### Bioinformatics analysis

The rat gene message transcripts of PPARγ were retrieved from the UCSC database (http://genome.ucsc.edu/), the promoter region sequences (2000 bp upstream of the transcription start site) of each transcript were obtained, and then the DNA motif of C/EBPα was obtained from the Jaspar website (http://jaspar.genereg.net). Possible binding sites were taken by predicting the matching result of the DNA motif to the sequence of the promoter region while referring to the species conservation of potential binding fragments. Primers were designed according to the sequence of possible binding sites for qPCR detection of ChIP products, and the qPCR primer sequences of possible binding sites are shown in Additional file 1: Data 2.

### Luciferase reporter assay

293 T cells stably expressing OE-Vector and OE-C/EBPα were cultured to the logarithmic phase, and 5 × 10^4^ cells were seeded per well of a 96-well plate one day before transfection. At least 2 replicate wells were plated for each well. 293 T cells were transfected with pGL3 empty vector, wild-type (WT)-PPARγ, mutant (MUT)-PPARγ and Renilla control vector using Lipofectamine 2000 after cell attachment. Fresh medium was replaced after 12 h of transfection. After 48 h of transfection, the cells were removed from the incubator and left at room temperature for 30 min. Then, 75 μl of Duo-Lite Luciferase assay reagent was added to 75 μl of culture, mixed well and incubated at room temperature for 10 min, and firefly luciferase luminescence was detected in an EnVision Multimode Microplate Reader (PerkinElmer). Then, 75 μl of Duo-Lite Stop & Lite detection reagent was added, and Renilla luciferase luminescence was detected after 10 min of incubation at room temperature. A dual luciferase reporter gene system assay was performed using the Duo-Lite Luciferase Assay System (Vazyme, Nanjing).

### Statistical analysis

All data are presented as the mean ± SD and were analysed with GraphPad Prism 9.0 (GraphPad Software, CA, USA). Unless otherwise stated, all experiments were repeated at least 3 times. The differences between groups were analysed by Student’s t test and one-way ANOVA. A value of *P* < 0.05 was considered statistically significant.

## Results

### C/EBPα and PPARγ levels were elevated in the SANFH model

**Fig. 1:**
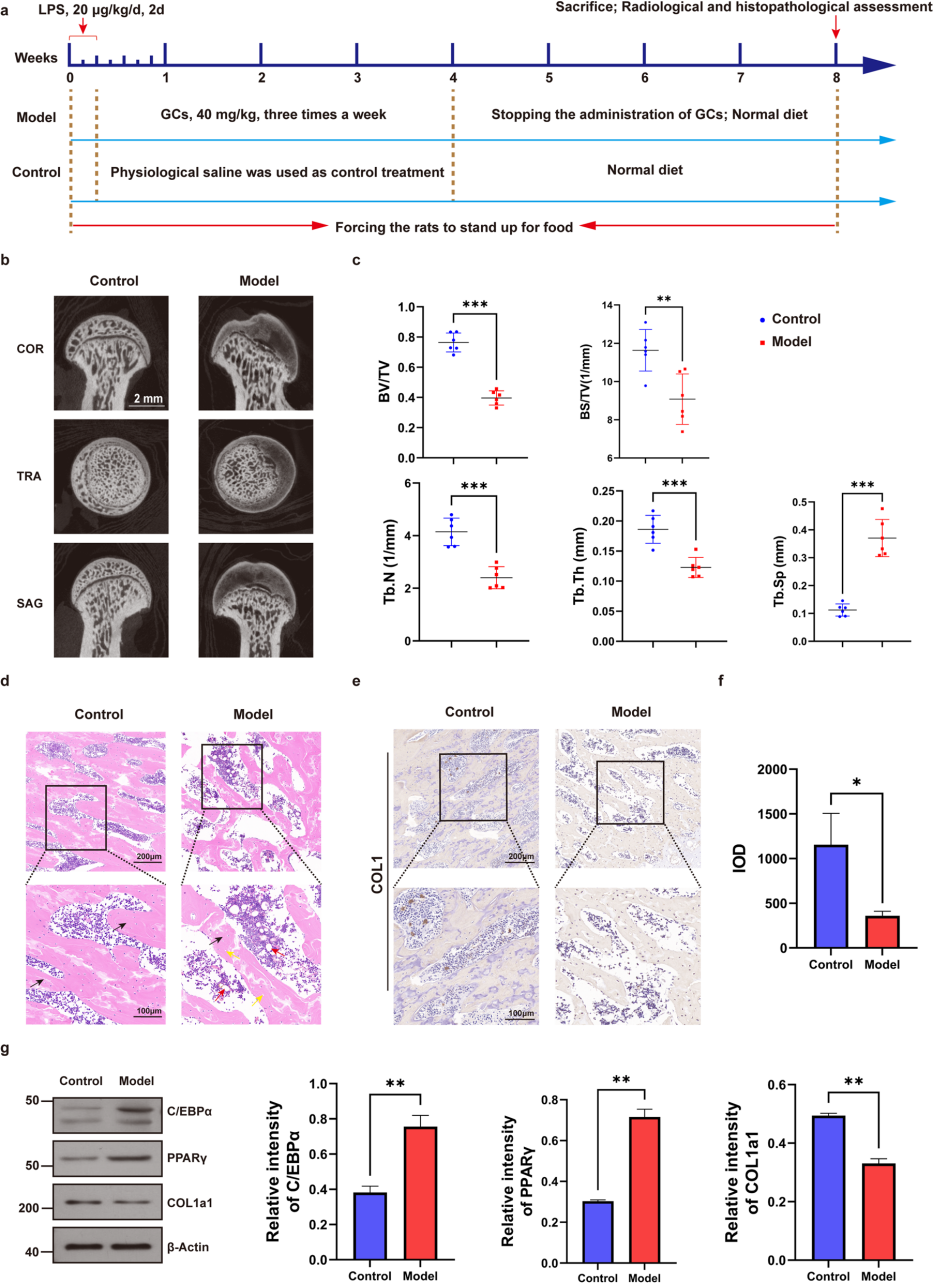
Establishment and evaluation of SANFH rat models. **a** Scheme of animal treatments. **b** The coronal (COR), transverse (TRA) and sagittal (SAG) sections of the femoral head were reconstructed by the micro-CT images in the control and model groups. **c** Quantitative analysis of related parameters of micro-CT. **d** H&E staining of the femoral head after decalcification, with black arrows indicating trabecular bone, red arrows indicating fat vacuoles, and yellow arrows indicating empty bone lacunae. **e, f** IHC staining of COL1 in the femoral head and quantitative analysis showed the IOD in the control and model groups. **g** The expression levels of femoral head-related proteins in the control and model groups were detected by western blotting, and β-actin was used for normalization and quantitative analysis by ImageJ. ***P < 0.001, **P < 0.01, *P < 0.05

Our data showed that the SANFH rat model was successfully established in vivo, as shown in Fig. 1. More bone loss and bone structure damage were observed by micro-CT in the model group than in the control group (Fig. 1b). In the analysis of relevant parameters, BV/TV, BS/TV, TB. N and TB. Th in the model group were significantly reduced, while TB. Sp was increased (Fig. 1c). H&E staining of femoral head sections showed that adipose tissue and enlarged adipocytes occupied the bone marrow cavity in the model group, with loose and thin bone trabeculae, disordered texture and more empty bone lacunae (Fig. 1d). IHC staining demonstrated that COL1 decreased significantly in the model group (Fig. 1e, f). Furthermore, we extracted total protein from femoral head samples for western blotting to detect the expression of adipogenesis- and osteogenesis-related genes, and the results showed that the expression of C/EBPα and PPARγ significantly increased and COL1a1 significantly decreased in the model group compared with the control group (Fig. 1g).

### C/EBPα inhibits osteogenic differentiation of BMSCs

**Fig. 2:**
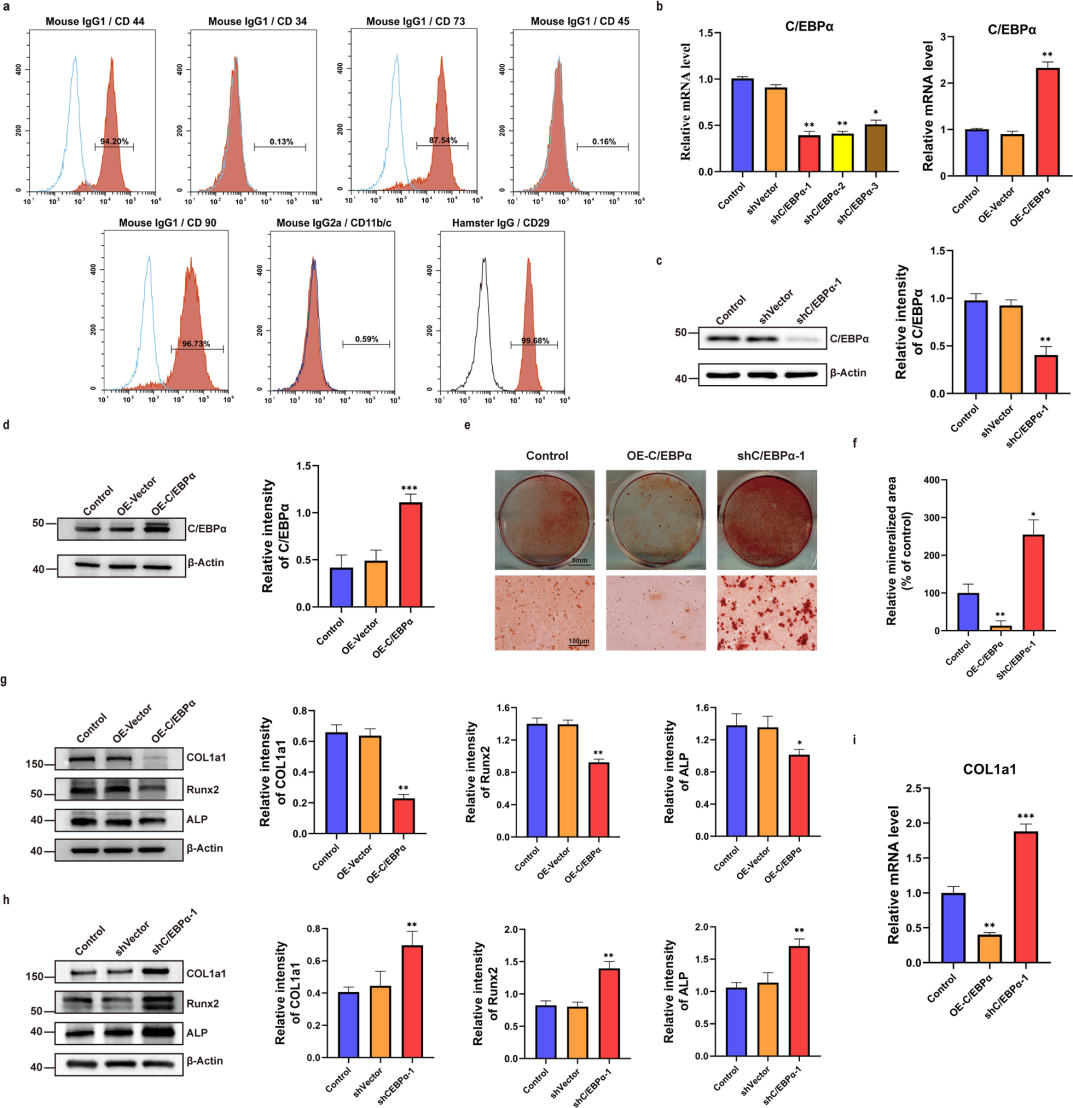
C/EBPα inhibits osteogenic differentiation of BMSCs. **a** The specific surface markers of BMSCs analysed by flow cytometry. **b**–**d** The establishment of knockdown or overexpression of C/EBPα determined by RT–qPCR and western blot analysis, respectively. **e, f** Alizarin red staining of osteogenic differentiation of BMSCs in each group and quantitative analysis by ImageJ. **g**–**i** The protein or mRNA levels of osteogenesis-related genes in BMSCs. ***P < 0.001, **P < 0.01, *P < 0.05

BMSCs were obtained from rat bone marrow by the differential adhesion method and were detected by flow cytometry at the third generation. The results showed that the surface markers anti-CD44, CD90, CD29 and CD73 were strongly positive and the differentiated associated markers anti-CD34, CD11b/c and CD45 were negative (Fig. 2a). Then, to verify the effect of C/EBPα on the osteogenic differentiation of BMSCs, BMSCs with overexpression of C/EBPα or knockdown of C/EBPα were constructed by lentivirus infection experiments. The infection efficiency observed by inverted fluorescence microscopy was above 90% (Additional file 1: Data 3), and high-purity BMSCs with OE-C/EBPα or shC/EBPα were obtained through puromycin drug screening (9 μg/ml, 4 d). The overexpression and knockdown efficiency of C/EBPα in BMSCs were analysed by RT–qPCR and western blot, and lentivirus carrying shC/EBPα-1 was ultimately selected as the knockdown tool (Fig. 2b-d). Next, we induced osteogenic differentiation of BMSCs with OE-C/EBPα and shC/EBPα-1 and evaluated calcium mineralization by alizarin red staining. The results showed that both the osteogenic differentiation ability and calcium deposition of BMSCs with OE-C/EBPα significantly decreased, while BMSCs with shC/EBPα-1 showed a strong potential for osteogenic differentiation (Fig. 2e, f). In addition, the protein expression levels of osteogenesis-related markers, including COL1a1, Runx2 and ALP, were significantly decreased in BMSCs with OE-C/EBPα, while the opposite results were observed in BMSCs with shC/EBPα-1 (Fig. 2g, h). The mRNA expression of COL1a1 was further detected by RT–qPCR, and the results were consistent with the above (Fig. 2i).

### C/EBPα regulates PPARγ transcriptional activity and promotes adipogenesis

**Fig. 3:**
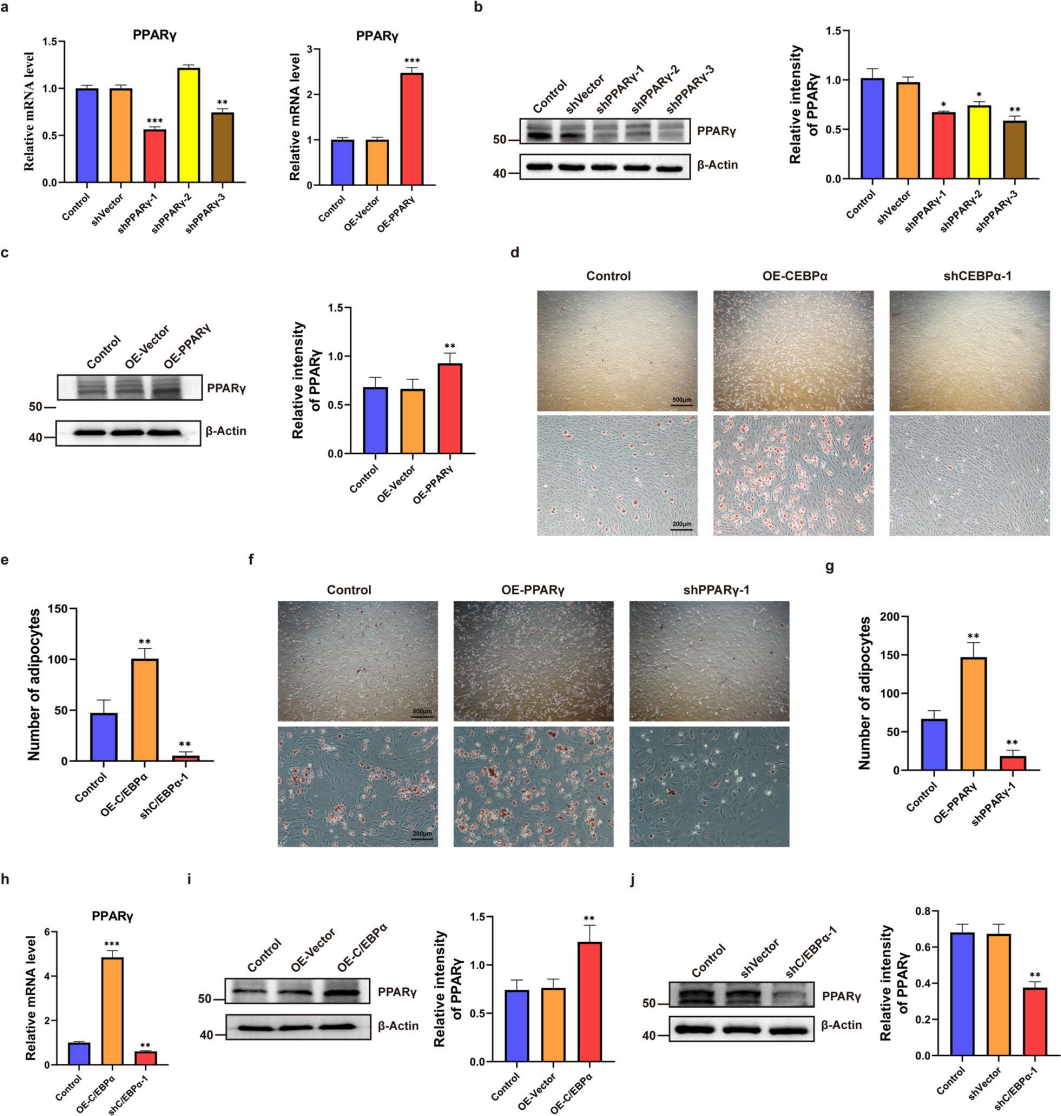
C/EBPα regulates PPARγ expression and promotes fat formation. **a**–**c** The establishment of PPARγ knockdown or overexpression determined by RT–qPCR and western blot analysis, respectively. **d**–**g** Oil red O staining images after inducing adipogenic differentiation of BMSCs in different groups. The number of adipocytes in each field (100×) was quantitatively analysed. **h**–**j** The mRNA and protein levels of PPARγ in BMSCs transfected with OE-C/EBPα and shC/EBPα-1. ***P < 0.001, **P < 0.01, *P < 0.05

To verify the effect of PPARγ on adipogenic differentiation of BMSCs from SD rats, PPARγ was overexpressed or knocked down, and the efficiency was determined by RT–qPCR and western blot analysis. A lentivirus carrying shPPARγ-1 was ultimately selected as a knockdown tool (Fig. 3a–c). Next, we induced adipogenic differentiation of BMSCs with overexpression and knockdown of PPARγ or C/EBPα. The results of oil red O staining showed that C/EBPα and PPARγ were positively associated with adipogenic differentiation, and knocking out either of them would lead to the failure of adipogenic differentiation (Fig. 3d–g). Furthermore, through RT–qPCR and western blot analysis, we found that there was a significant positive correlation between C/EBPα and the expression of PPARγ (Fig. 3h–j).

**Fig. 4:**
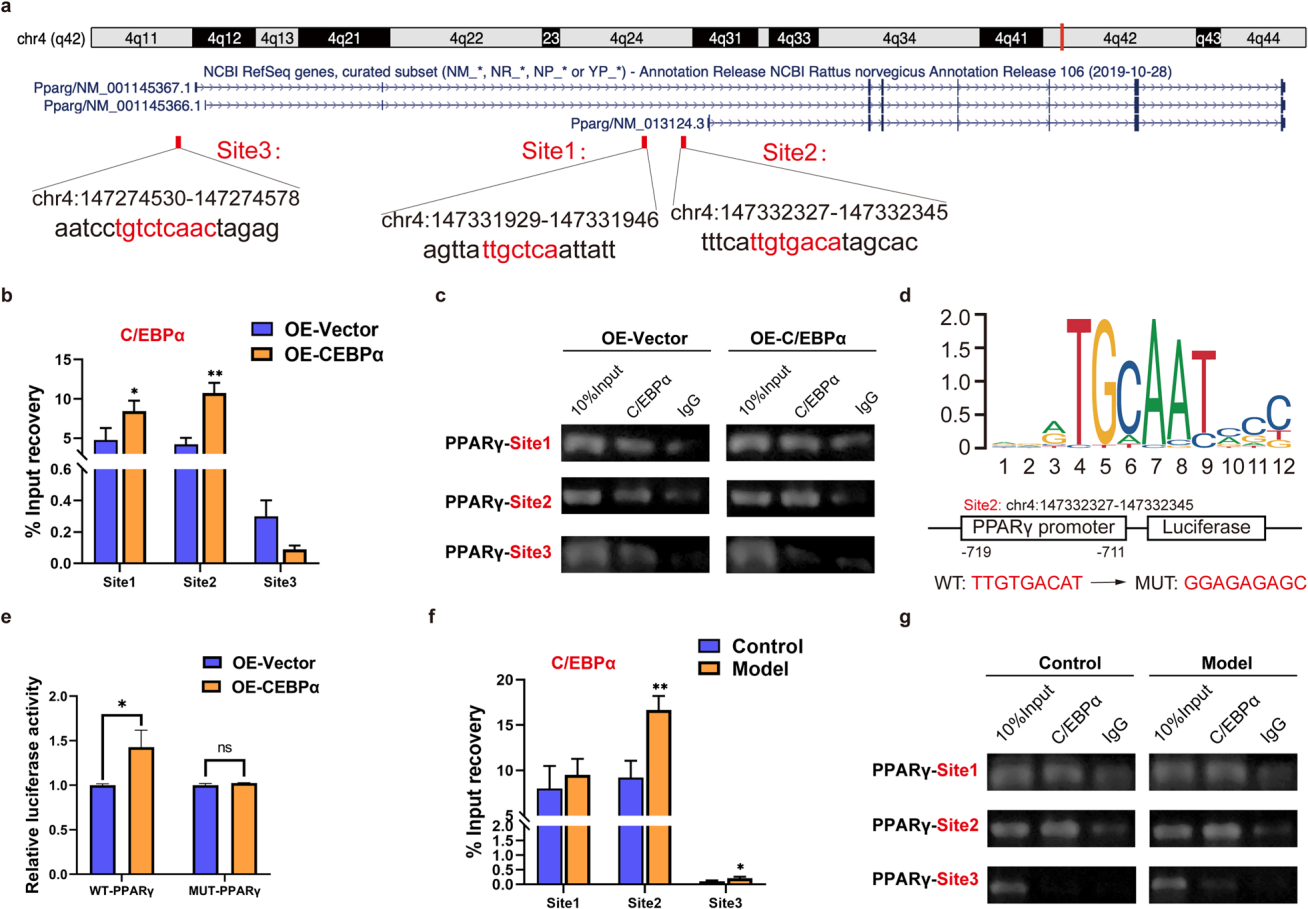
C/EBPα regulates the transcriptional activity of the PPARγ promoter. **a** Bioinformatic prediction results of potential binding sites of C/EBPα in the PPARγ promoter region. **b, c** ChIP analysis of PPARγ promoter binding sites in BMSCs with OE-Vector and OE-C/EBPα and agarose gel electrophoresis of ChIP–qPCR products. **d** The potential binding sites of C/EBPα in the promoter of the PPARγ gene and the structural diagram of the luciferase reporter of WT-PPARγ or MUT-PPARγ binding sites. **e** Analysis of relative luciferase activity. **f, g** ChIP assay results of C/EBPα binding at the PPARγ promoter region in femoral head tissues of SD rats in the normal and model groups. The ChIP–qPCR products were subjected to agarose gel electrophoresis. **P < 0.01, *P < 0.05, ns = not significant

Next, we investigated the potential mechanism by which the transcription factor C/EBPα regulates PPARγ expression. Bioinformatics analysis was conducted and predicted that there were three potential binding sites of C/EBPα within 2 kb upstream of the PPARγ transcription initiation site (Fig. 4a). The ChIP assay revealed that C/EBPα was significantly enriched at two sites (site 1 and site 2) compared with the control group, especially site 2, which indicated that C/EBPα was bound up with the PPARγ promoter at site 2 (Fig. 4b, c). To further verify the results, a luciferase reporter assay was performed in 293 T cells (Additional file 1: Data 4). The results showed that OE-C/EBPα significantly increased the luciferase activity of WT-PPARγ (insertion site 2) compared with the empty vector or MUT-vector. However, changes in luciferase activity were eliminated in MUT-PPARγ (site 2 mutation) (Fig. 4d, e). In summary, these results suggested that C/EBPα can directly regulate the activity of the PPARγ promoter, thereby jointly controlling the process of adipogenesis in vitro. Furthermore, the femoral heads in the control and model groups were collected, and a ChIP assay was conducted to verify this process in vivo (Additional file 1: Data 5). Compared with the control group, C/EBPα in the model group significantly bound at two sites (site 2 and site 3) in the PPARγ promoter region, especially site 2 (Fig. 4f, g), which is partially consistent with the in vitro results.

### Histone acetylation of PPARγ mediated epigenetic mechanisms in SANFH

**Fig. 5:**
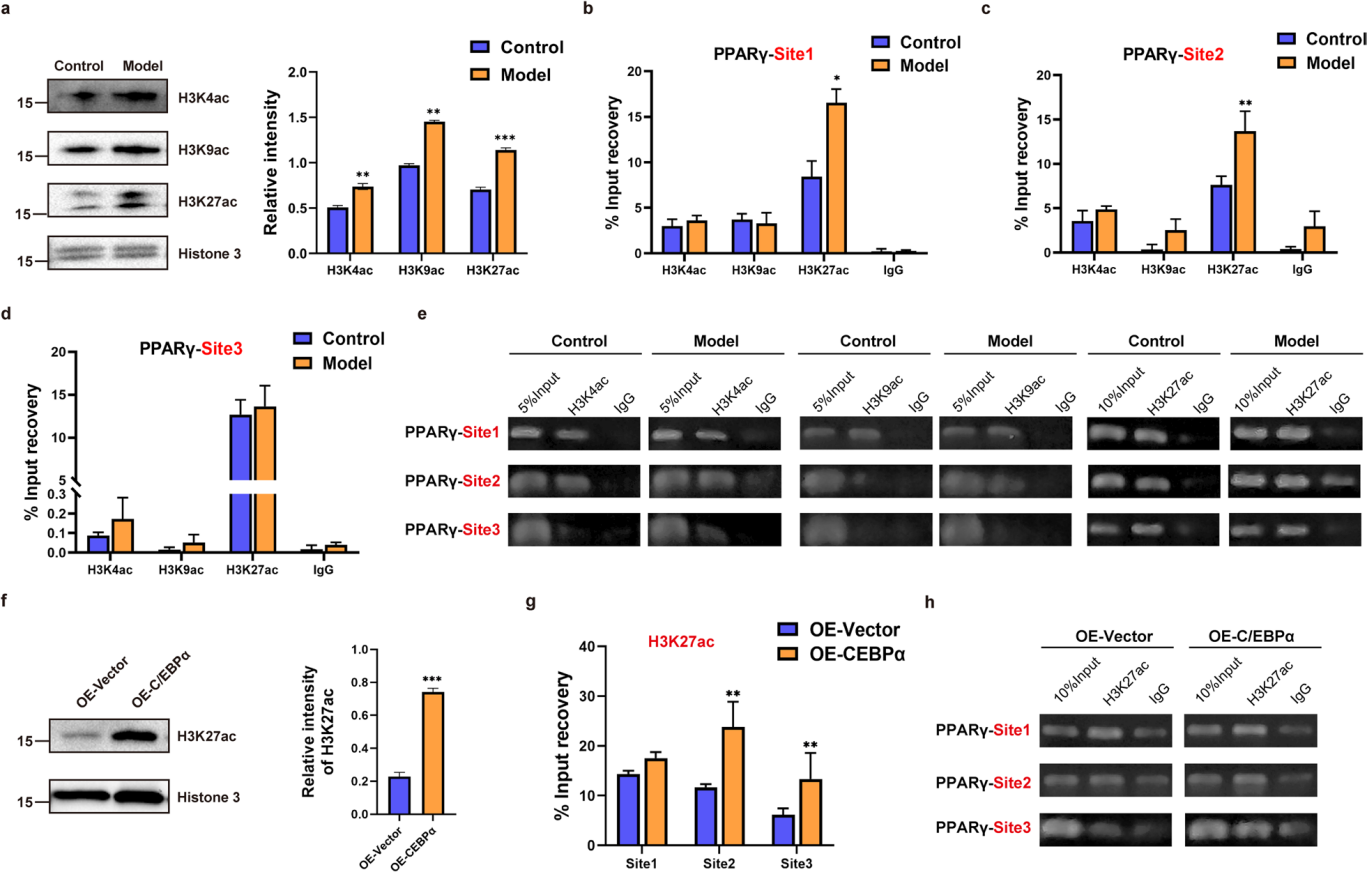
Histone acetylation of the PPARγ promoter in SANFH. **a** The expression of three acetylated histones in the control and model groups, with histone 3 as a standard. **b**–**d** ChIP–qPCR analysis of three acetylated histones in the PPARγ promoter region in the control and model groups. **e** Agarose gel electrophoresis of the ChIP–qPCR products. **f** The expression of H3K27ac in BMSCs with OE-Vector and OE-C/EBPα; histone 3 served as the standard. **g, h** ChIP–qPCR analysis of three sites of H3K27ac in the PPARγ promoter region; the nonspecific IgG group was omitted for display. ***P < 0.001, **P < 0.01, *P < 0.05

Furthermore, we explored the regulatory mechanism of continuous PPARγ expression, and we observed that osteonecrosis continued to progress after stopping the administration of GCs in SANFH. Through the nucleocytoplasmic isolation experiment and western blot analysis of femoral head tissue, the levels of acetylated histone H3K4 (H3K4ac), acetylated histone H3K9 (H3K9ac) and acetylated histone H3K27 (H3K27ac) were significantly increased in the model group (Fig. 5a). ChIP–qPCR analysis demonstrated that only H3K27ac was significantly more enriched at site 1 and site 2 of the PPARγ promoter region in the model group than in the control group (Fig. 5b–e). Thus, it can be reasonably inferred that histone H3K27 acetylation is conducive to the transcription and sustained expression of PPARγ and plays an important role in SANFH. Similarly, in vitro, the nuclear protein of BMSCs with OE-Vector and OE-C/EBPα was extracted for western blotting. Compared with the OE-Vector group, the expression of H3K27ac in the OE-C/EBPα group was significantly increased (Fig. 5f). ChIP–qPCR analysis showed that H3K27ac was significantly enriched at site 2 of the PPARγ promoter region in the OE-C/EBPα group, and the binding of H3K27ac at site 3 was also increased (Fig. 5g, h). Taken together, these results suggest that C/EBPα can promote acetylation of histone H3K27 in the PPARγ promoter region, mediating PPARγ activation and continuous expression and ultimately leading to fat accumulation and SANFH.

### Histone acetylation of PPARγ is crucial to adipogenic differentiation of BMSCs in vitro

**Fig. 6:**
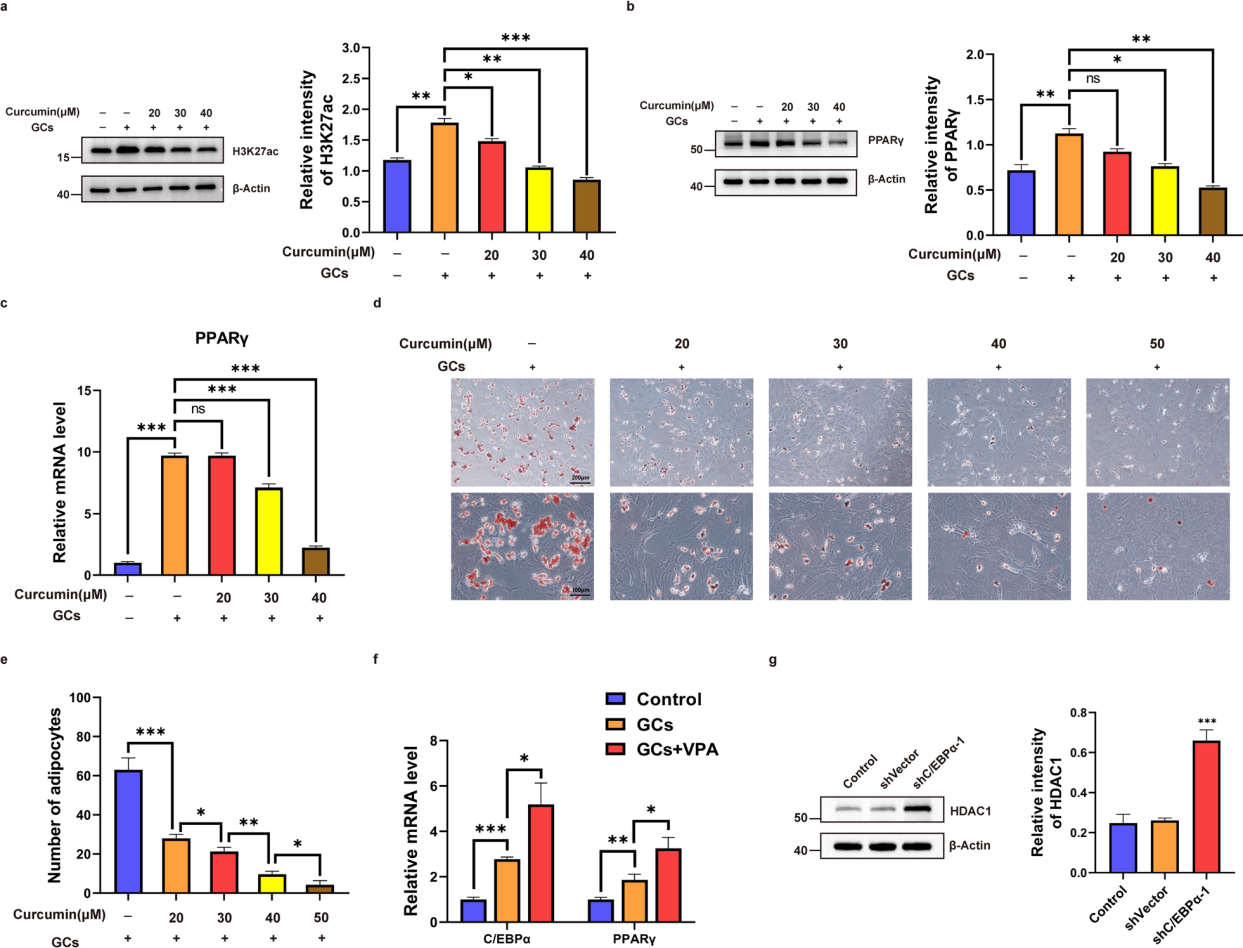
Histone acetylation of PPARγ is crucial to adipogenic differentiation of BMSCs in vitro. **a**–**c** The expression levels of H3K27ac and PPARγ in BMSCs in different groups detected by western blot or RT–qPCR. **d, e** Images and quantitative analysis of oil red O staining in groups with different concentrations of curcumin. **f** The mRNA expression levels of C/EBPα and PPARγ. **g** The protein expression level of HDAC1 in BMSCs transfected with shC/EBPα-1. ***P < 0.001, **P < 0.01, *P < 0.05, ns = not significant

Curcumin, a typical histone acetylase inhibitor, is widely used for in vitro and in vivo studies [37]. To fully verify the effect of PPARγ acetylation on adipogenic differentiation of BMSCs, we set up three groups: control, GC and GC + curcumin with different gradient concentrations of curcumin (20, 30, 40 μM) acting on the BMSCs in vitro. After 3 days, the expression levels of H3K27ac and PPARγ were detected by RT–qPCR or western blot. The results showed higher expression levels of H3K27ac and PPARγ in the GC-treated group, but the expression levels of H3K27ac and PPARγ gradually decreased with successive increases in the concentration of curcumin (Fig. 6a–c). Curcumin reduced the level of H3K27 acetylation in BMSCs and downregulated the expression of PPARγ. Then, BMSCs were induced to adipogenic differentiation in GC-supplemented medium and simultaneously treated with a gradient of curcumin concentrations (0, 20, 30, 40, 50 μM). Oil red O staining showed fewer positive areas as the curcumin concentration increased (Fig. 6d, e). To further explain the positive role of histone acetylation in adipogenic differentiation, BMSCs were treated with a selective HDAC1 inhibitor, valproic acid (VPA) [38, 39], to upregulate histone acetylation levels in vitro. Then, the control, GC and GC + VPA groups were established, and the expression of C/EBPα and PPARγ in BMSCs was detected by RT–qPCR. The results revealed that VPA could significantly increase the expression of C/EBPα and PPARγ in BMSCs in the presence of GCs (Fig. 6f). These results suggested that histone acetylation of PPARγ is crucial to adipogenic differentiation of BMSCs induced by GCs. Interestingly, upregulated expression of HDAC1 was observed in BMSCs with shC/EBPα-1, indicating a negative correlation between C/EBPα and HDAC1 (Fig. 6g).

### Curcumin rescues SANFH by inhibiting PPARγ expression in vivo

**Fig. 7:**
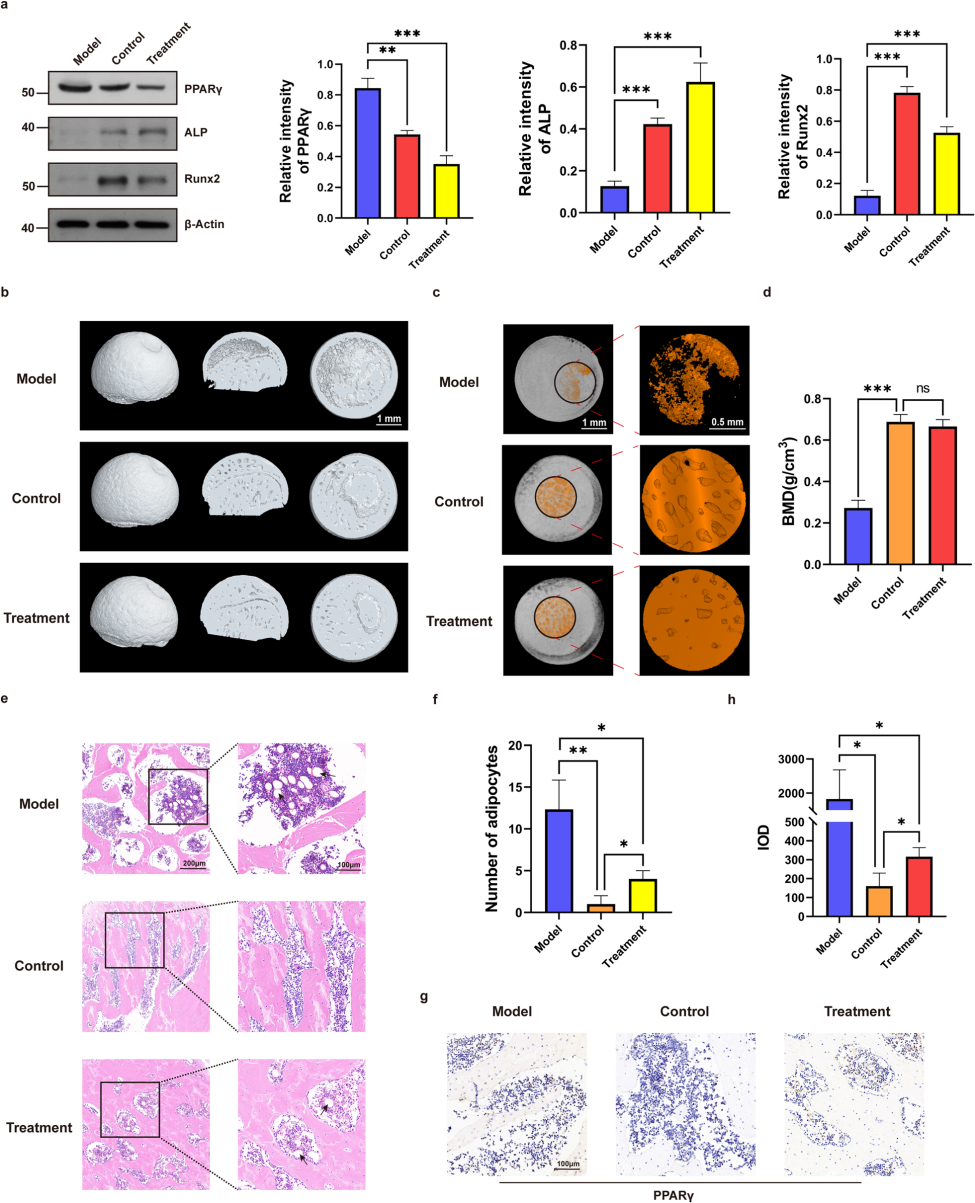
Curcumin rescues SANFH by inhibiting PPARγ expression in vivo. **a** Expression levels of PPARγ, ALP and Runx2 in the femoral heads of each group as detected by western blot. **b** 3D reconstructed images of the femoral head. **c** Circular region of interest (*r* = 0.75 mm) in images for evaluating internal bony structure and bone marrow cavity. **d** BMD analysis of femoral heads in each group. **e, f** H&E staining for assessing the levels of intramedullary fat in each group, with black arrows indicating fat vacuoles. **g, h** IHC analysis of PPARγ expression in each group. ***P < 0.001, **P < 0.01, *P < 0.05, ns = not significant

To better study the performance of curcumin as an acetylase inhibitor in vivo, the control, model and curcumin intervention (treatment) groups were used to evaluate the ability of curcumin to rescue SANFH in SD rats. Compared with the model group, curcumin significantly reduced the expression of PPARγ in the treatment group and prevented the decrease in the expression of the osteogenesis-related genes ALP and Runx2 (Fig. 7a). Then, micro-CT imaging and 3D structure reconstruction were conducted to assess bone formation and remodelling. Compared with the model group, early curcumin intervention significantly improved bone loss and destruction, and quantitative microstructure parameters showed that the BMD of the treatment group basically reached the level of the control group. However, the area of the medullary cavity of the femoral head was reduced for unknown reasons (Fig. 7b–d). Finally, H&E and IHC staining were performed to determine the levels of intramedullary fat and PPARγ expression in tissue, respectively. Less intramedullary fat was observed in the treatment group than in the model group but was still slightly higher than that in the control group (Fig. 7e, f). As shown by the IHC staining images, the content of PPARγ in the treatment group was significantly reduced but slightly higher than that in the control group (Fig. 7g, h). These results suggested that early curcumin intervention could reduce intramedullary fat production in the femoral head by inhibiting PPARγ expression in vivo and thus slow the progression of SANFH.

## Discussion

SANFH, a common and rapidly disabling disease, is usually caused by the treatment of GCs in many nonorthopaedic diseases. Most patients will develop femoral head collapse within 2–3 years and eventually hip dysfunction [40, 41], resulting in inestimable labour and economic loss for the patients' families and the whole society. Strangely, although BMSCs have strong proliferative potential, it is still difficult to reverse the disease progression of SANFH after pathogenic factors (i.e. the intake of GCs) are removed [42]. In reviewing the literature, no data were found to explain this phenomenon. In this study, SD rats received intermittent high doses of GC during the first 4 weeks, followed by GC withdrawal for an additional 4 weeks. The results suggest that the SANFH model was successfully established in vivo.

Increasing evidence suggests that SANFH is a disease associated with abnormal differentiation of BMSCs [15, 43–45], and the treatment of SANFH with BMSC transplantation in animal models or human experiments has achieved excellent results [46–48]. The current in vivo and in vitro studies found that GCs significantly inhibited the osteogenic differentiation of BMSCs and promoted the adipogenic differentiation of BMSCs. The intramedullary fat of the femoral head accumulated gradually, which lead to an increase in intraosseous pressure, a decrease in arterial perfusion and the obstruction of venous reflux, resulting in irreversible necrosis of the femoral head.

Adipogenic differentiation is a tightly regulated process orchestrated by a number of transcription factors. Prior studies have noted the importance of C/EBPα and PPARγ in adipogenesis and lipid accumulation, respectively [49, 50], but there is a lack of a specific regulatory mechanism of C/EBPα targeting the PPARγ signalling pathway in adipogenic differentiation of BMSCs. In the SANFH model, C/EBPα and PPARγ were highly expressed in the femoral head. C/EBPα was verified to significantly inhibit bone repair by preventing osteogenic differentiation of BMSCs in vitro. To explore the relationship between C/EBPα and PPARγ, lentivirus-mediated gene knockdown and overexpression assays were performed, and we proved the positive regulatory effect of C/EBPα on PPARγ in BMSCs by RT–qPCR and western blotting. Furthermore, ChIP–qPCR and luciferase reporter assays also concluded that the transcription factor C/EBPα could directly enhance the transcriptional activity of the PPARγ promoter region. In addition, these conclusions were consistent with findings in vivo.

Epigenetic research explores heritable changes in gene expression without changing nucleotide sequences. It plays an important role in growth, development and disease evolution [51–53]. This study confirmed that epigenetic factors are associated with the occurrence of SANFH, which may to a certain extent explain why femoral head necrosis continues to progress after removing external factors (i.e. stopping GC therapy). The balance of histone acetylation and deacetylation is critical for the regulation of genes and epigenetic control, and some studies have shown that acetylation of histones of PPARγ is related to adipogenesis [54]. To test our hypothesis, we first explored the epigenetic regulation mode of PPARγ in vivo. As expected, the H3K27ac modification level of the PPARγ promoter region in the femoral head was significantly increased in the SANFH model of SD rats, which could promote the sustained and stable expression of PPARγ. Moreover, BMSCs were also studied in vitro, and consistent results were observed in BMSCs with OE-C/EBPα. These findings further support the idea that PPARγ histone acetylation is involved in the adipogenic differentiation of BMSCs and the occurrence of SANFH. Curcumin, a natural active component of turmeric, has been proven to have great potential in regulating epigenetics [37, 55]. Its ability to inhibit histone acetylase activity was employed to verify the effects of intervening in PPARγ acetylation on the adipogenic differentiation of BMSCs and SANFH. The results of this study indicated that curcumin could inhibit PPARγ expression and adipogenic differentiation of BMSCs in vitro. Meanwhile, curcumin can also reduce intramedullary lipogenesis of the femoral head and prevent the onset of SANFH in vivo. Another important finding was that HDAC1 may be involved in the regulation of PPARγ histone acetylation. The expression level of PPARγ in BMSCs treated with VPA was increased, and HDAC1 was highly expressed in BMSCs with shC/EBPα-1, indicating that C/EBPα may upregulate the level of PPARγ acetylation by inhibiting HDAC1, thus promoting the continuous expression of PPARγ. Further research should be undertaken to investigate the specific regulatory mechanisms in the future.

However, there are still some limitations in this study. First, due to the characteristics of human upright walking and species differences, an animal model of SD rats may not exactly match human SANFH. Second, as a natural product, curcumin shows a diverse range of pharmacological effects and is not a highly selective inhibitor of histone acetylase, although that is not expected to affect the interpretation of the experimental results in this study. We speculate that the reason for the reduced area of the medullary cavity of the femoral head in the treatment group (Fig. 7b, c) may be attributed to curcumin. Finally, plasmid transfection was inefficient in BMSCs due to the characteristics of the primary cells, and 293 T cells were chosen as classical tool cells for luciferase reporter assays.

## Conclusion

Taken together, our results demonstrate that C/EBPα mediates adipogenic differentiation of BMSCs and participates in the onset of SANFH by targeting the PPARγ signalling pathway. The histone acetylation of PPARγ is an important intermediate process in SANFH. These findings enrich the epigenetic mechanism of pathological damage in SANFH and provide new ideas for the treatment strategy of SANFH.

## Supplementary Information

**Additional file 1**. Supplementary Data 1-5.

## Data Availability

The data sets used during the current study are available from the corresponding authors on reasonable request.

## Abbreviations

ALP: Alkaline phosphatase, biomineralization associated
BMD: Bone mineral density
BMSCs: Bone marrow mesenchymal stem cells
BV/TV: Bone volume/tissue volume
BS/TV: Bone surface area/tissue volume
ChIP: Chromatin immunoprecipitation
C/EBPα: CCAAT/enhancer-binding protein α
COL1a1: Collagen type I, alpha 1 chain
COR: Coronal
GC: Glucocorticoid
GR: Glucocorticoid receptor
H3K4ac: Acetylated histone H3K4
H3K9ac: Acetylated histone H3K9
H3K27ac: Acetylated histone H3K27
H&E: Hematoxylin and eosin
HDAC1: Histone deacetylase 1
IHC: Immunohistochemistry
IOD: Integrated optical density
micro-CT: Microcomputed tomography
MUT: Mutant
OE: Overexpression
PPARγ: Peroxisome proliferator-activated receptor γ
PVDF: Polyvinylidene fluoride
SAG: Sagittal
SANFH: Steroid-induced avascular necrosis of the femoral head
SD: Sprague Dawley
shRNA: Short-hairpin RNA
TB. N: The number of trabeculae
TB. Th: Trabecular thickness
TB. Sp: Trabecular separation
TRA: Transverse
VPA: Valproic acid
WT: Wild type
