# Supplementary material for: C/EBPα regulates the fate of bone marrow mesenchymal stem cells and steroid-induced avascular necrosis of the femoral head by targeting the PPARγ signalling pathway
Source: Stem Cell Res Ther. 2022 Jul 26;13:342. doi: 10.1186/s13287-022-03027-3 (PMC9327281; doi:10.1186/s13287-022-03027-3)

**Title page**

**C/EBPα regulates the fate of bone marrow mesenchymal stem cells and steroid-induced avascular necrosis of the femoral head by targeting the PPARγ signalling pathway**

Ping Duan^1^, Hanyu Wang^1^, Xinzeyu Yi^1^, Hao Zhang^1^, Hui Chen^1^,

Zhenyu Pan^1*^

**Affiliations:**

^1^ Department of Orthopedics Trauma and Microsurgery, Zhongnan Hospital of Wuhan University, Wuhan 430071, China.

***** **Corresponding author:**

Zhenyu Pan

Department of Orthopedics Trauma and Microsurgery, Zhongnan Hospital of Wuhan University, Wuhan 430071, China

Tel: +86 15337200318

E-mail: soloistp@sina.com

**Supplementary Data 1**

**Primers for quantitative RT-PCR**

| Gene | Sequence |
| --- | --- |
| ACTB-F | AGATCAAGATCATTGCTCCTCCT |
| ACTB-R | ACGCACCTCAGTAACAGTCC |
| C/EBPα-F | GACCATCCGCCTTGTGTGTA |
| C/EBPα-R | CTGACATTGCACAAGGCACC |
| PPARγ-F | AGCCTTCAAACTCCCTCATGG |
| PPARγ-R | GAGACATCCCCACAGCAAGG |
| COL1a1-F | GATGGACTCAACGGTCTCCC |
| COL1a1-R | CGGCCACCATCTTGAGACTT |
| HDAC1-F | AGGGAGAAGGAGGTCGCAAG |
| HDAC1-R | ACTTGGAGAGAAGATGGAGCTG |
| ALP-F | CACTATGTCTGGAACCGCACT |
| ALP-R | GGGTCAGTCAGGTTGTTCCG |
| aP2-F | TGGGGACCTGGAAACTCGT |
| aP2-R | AGTCCCCTTCTACGCTGATG |

**Supplementary Data 2**

**Primers were designed according to the sequence of** **possible binding sites**

| Gene Name | Prediction site |  | Primer |
| --- | --- | --- | --- |
| PPARγ promoter | Site 1 | F | GATGCCAGAGATAGCACATAGAAC |
|  |  | R | ATCACAAACCCAGGACACACTA |
|  | Site 2 | F | CAGTGAGCATTTTCCCATTGAGT |
|  |  | R | CCTGATACCCCCAAATGGAATAAC |
|  | Site 3 | F | TGACTGTCTAGGTGACTGGGT |
|  |  | R | TTTGTCTGTCACGCTCCGTC |

**Supplementary Data 3**

**Images of BMSCs infected with lentivirus (Under the same field of view)**


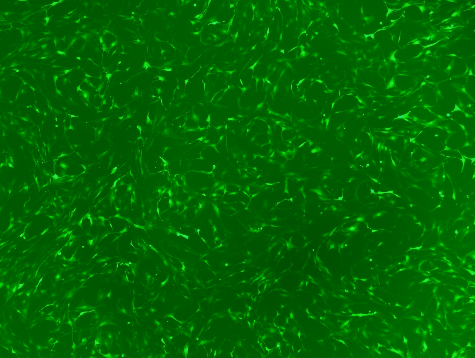

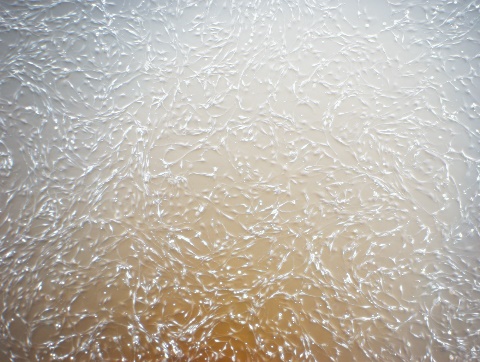


40X (Bright field) 40X (GFP green fluorescence)


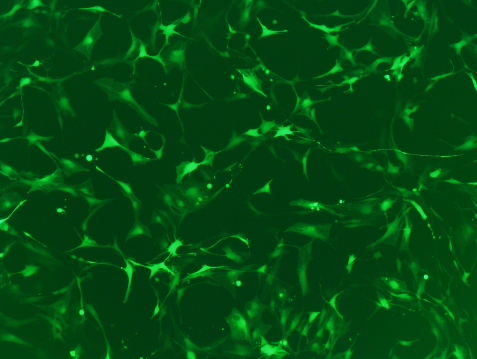

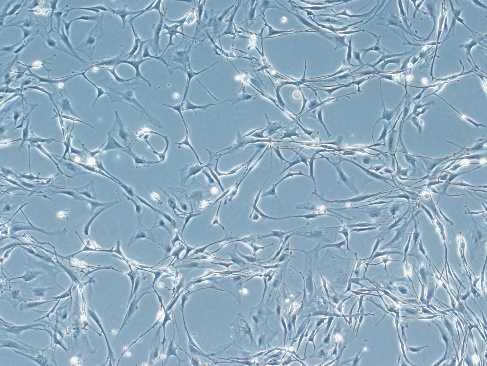


100X (Bright field) 100X (GFP green fluorescence)

**Supplementary Data 4**

**4a Peak map of mutation site**

**
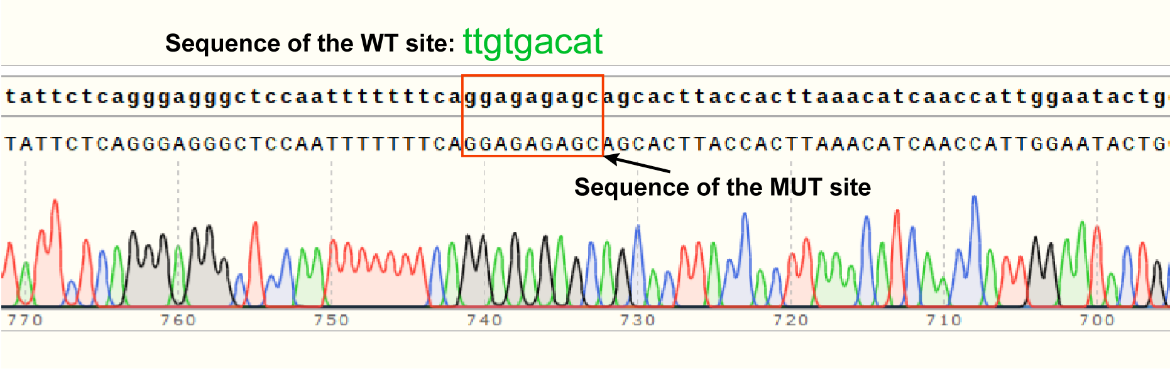
**

**4b The PCR results of overexpression of C/EBPα were in 293T**


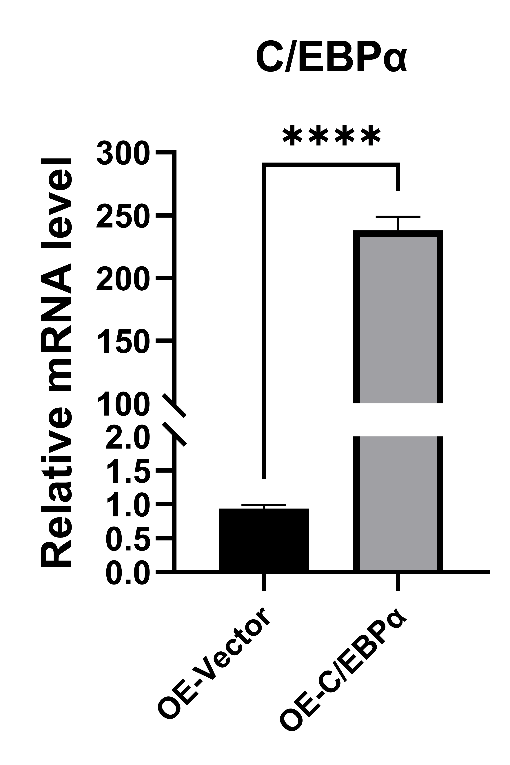


**Supplementary Data 5**

**5a Western blotting analysis of nuclear protein in femoral head**

**
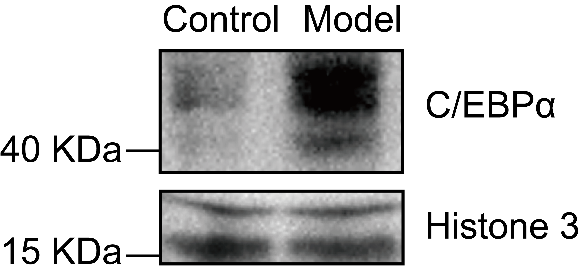
**

**5b Electrophoretogram of DNA after ultrasonic fragmentation in** **femoral head**


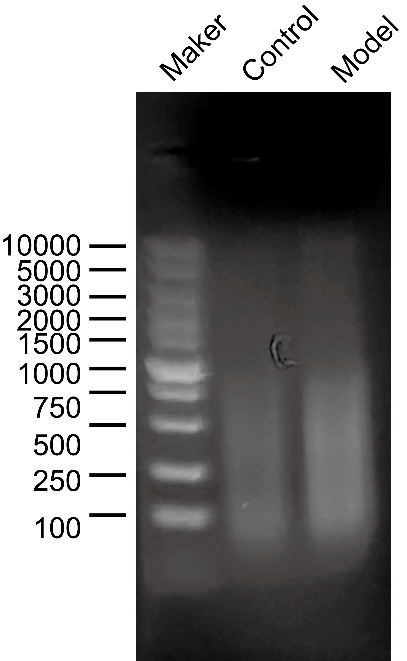

Supplement: Supplementary file 1 — Additional file 1. Supplementary Data 1-5. [file 13287_2022_3027_MOESM1_ESM.docx]
